# Factors associated with length of stay and the risk of readmission in an acute psychiatric inpatient facility: a retrospective study

**DOI:** 10.3109/00048674.2011.585452

**Published:** 2011-06-30

**Authors:** Jianyi Zhang, Carol Harvey, Carol Andrew

**Affiliations:** North West Area Mental Health Service, Broadmeadows, Victoria, Australia; Alfred Hospital, Commercial Road, Prahran, Victoria 3004, Australia. Email: j.zhang@alfred.org.au; Department of Psychiatry, University of Melbourne and North West Area Mental Health Service, Coburg, Victoria, Australia; North West Area Mental Health Service, Coburg, Victoria, Australia

**Keywords:** mental health, hospital readmission, length of stay, patient admission

## Abstract

**Objective:**

This study was to investigate factors influencing the length of stay and predictors for the risk of readmission at an acute psychiatric inpatient unit.

**Method:**

Two comparative studies were embedded in a retrospective cross-sectional clinical file audit. A randomly selected 226 episodes of admissions including 178 patients during a twelve-month period were reviewed. A total of 286 variables were collected and analysed. A case control study was employed in the study of length of stay. A retrospective cohort study was used to investigate the predictors for the risk of readmission.

**Results:**

Logistic regression analyses showed that 10 variables were associated with length of stay. Seclusion during the index admission, accommodation problems and living in an area lacking community services predicted longer stay. During the follow-up period 82 patients (46%) were readmitted. Cox regression analyses showed 9 variables were related to the risk of readmission. Six of these variables increased the risk of readmission, including history of previous frequent admission, risk to others at the time of the index admission and alcohol intoxication. More active and assertive treatment in the community post-discharge decreased the risk of readmission.

**Conclusions:**

Length of stay is multifactorially determined. Behavioural manifestations of illness and lack of social support structures predicted prolonged length of stay. Good clinical practice did not necessarily translate to a shorter length of stay. Therefore, length of stay is predictable, but not readily modifiable within the clinical domain. Good clinical practice within the community following discharge likely reduces the risk of readmission. Quality of inpatient care does not influence the risk of readmission, which therefore raises a question about the validity of using the rate of readmission as an outcome measure of psychiatric inpatient care.

In the last few decades, western countries went through a process of deinstitutionalization of psychiatric care. There has been a signifi cant reduction of beds in psychiatric services with the closure of old psychiatric hospitals and the locus of care has been switched to alternative services in the community [1-4]. With deinstitutionalization, acute inpatient admission has become an important therapeutic option for severely ill psychiatric patients. However, difficulty in acute psychiatric bed access has been reported to be an ongoing problem. This difficulty in bed access has been viewed mainly as the result of bed blocking by ‘inappropriate admissions’ and lack of alternative services in the community [5-7].

The Auditor General of Victoria stated in his report in 2002: ‘It is becoming increasingly difficult for people to gain access to acute inpatient services and a very high level of symptom severity is necessary to gain access to beds’ [8]. Difficulty in psychiatric bed access has a ‘flow-on’ impact on the rest of the public health system, especially creating pressure on emergency departments (EDs) at general hospitals [9]. A similar situation exists internationally; for example, the 2007 annual meeting of the American Medical Association's House of Delegates passed a resolution to call for urgent attention on ‘the national scope of the problem of psychiatric bed availability’ and its impact on the nation's emergency departments [10]. Despite an increased number of acute psychiatric beds over the past few years in Victoria, the problem with bed availability remains a significant issue in the public mental health system. This problem has undermined public confidence in the mental health system and caused a strong dissatisfaction among consumers and carers [8,11].

A few factors have been linked to bed availability. Length of stay (LOS), readmission rate and inappropriate admissions are the main factors [5,12]. Available studies were either poorly designed [13], outdated or only looking into variables that are unlikely to be changed without significant change of social policy, for instance employment state, socioeconomic deprivation, and accommodation [5,14]. There has been little substantial relevant research conducted in Australia, although a recent study conducted in a West Australian private psychiatric hospital reported that hospital outcome assessed by patient-reported symptomatic improvement predicted readmissions [15]. Most findings of these studies are not applicable in a Victorian public mental health setting, as LOS and psychiatric inpatient admission are strongly related to the structure of the mental health system and cultural factors [16-18].

The primary objectives of this study were to investigate factors associated with LOS and predictors for the risk of readmission to an acute psychiatric unit in an Australian metropolitan catchment area. Other objectives were to profile patients admitted to an acute psychiatric ward in order to understand the current status of acute bed usage. This study had a focus of investigating variables that might be modifiable through change of clinical practice and service delivery.

## Method

### Study setting

North West Area Mental Health Service (NWAMHS) is an integrated adult psychiatric service. NWAMHS is part of North Western Mental Health Program (NWMHP). NWAMHS serves the north western metropolitan part of Melbourne. This area stretches from inner urban to industrial and suburban outskirts of Melbourne. It is a socially and economically disadvantaged area with an Index of Relative Social and Economic Disadvantage (IRSED) score of 972, making it the third most deprived catchment area of the 22 AMHS in Victoria [19]. Further, the northern part of the catchment area (Local Government Area (LGA) of Hume) is more deprived than the southern part (LGA of Moreland). There are two continuing care teams based in Coburg (Moreland) and Broadmeadows (Hume). The continuing care team is a community based psychiatric service that provides psychiatric treatment and follow-up to registered patients. The inpatient facility of NWAMHS is located in Broadmeadows. The Broadmeadows Inpatient Psychiatric Unit (BIPU) is a 25-bed acute adult psychiatric inpatient facility. This study was approved by the NWMHP Mental Health Research and Ethics Committee.

### Study design

In broad terms, this project included two comparison studies embedded in a retrospective cross-sectional file audit. A 12-month period from 1 July 2004 to 30 June 2005 was defined as the study period. There were a total of 621 admissions to BIPU during this period. Incomplete admissions were excluded from the study. Incomplete admission implies patients were transferred to BIPU from other acute psychiatric facilities or transferred to other acute psychiatric facilities from BIPU. If a patient died during an episode of admission, the episode of admission was also considered as an incomplete admission. A total of 524 admissions involving 407 patients met the inclusion criteria after the exclusion criteria were applied. We randomly selected 249 admissions involving 196 patients; 18 patients' files including 23 admissions (9%) were missing; 226 episodes of admissions, involving 178 patients, were included in the data analysis. A minimum sample size of 35 admissions or patients was required to achieve 80% statistical power to detect 30% difference between exposure and non-exposure groups.

A data collection instrument was designed to include three categories of variables: 18 sociodemographic variables, 212 clinical variables and 56 clinical practice or system variables. The main sociodemographic variables included age, gender, ethnicity, migrant status, marital status, accommodation, and employment status. The main clinical variables included previous history, duration and severity of illness, diagnosis, mental state examination (MSE), treatment, Health of the Nation Outcome Scale (HoNOS) score [20], reason for admission and further stay. Clinical practice variables included legal status, individual service plan (ISP) [21], carer's involvement, treating team issues. The clinical and system variables also included indicators of the quality of care, for instance service delivery and pharmacological intervention. A copy of the full data collection instrument is available from the corresponding author on request.

Comparison study 1 was to investigate factors associated with prolonged length of stay (LOS). Prolonged LOS was defined as LOS equal to or more than the median LOS during the year surveyed. The median LOS was 12 days in the sample population. The design of this study was similar to a case-control study. Cases were defined as episodes of admission with prolonged LOS. Controls were defined as episodes of admission with LOS less than the median LOS. Outcomes were defined as LOS and exposures were defined as variables listed in the data collection instrument. A total of 226 admissions were included in this study; 114 admissions were ‘cases’ and 112 admissions were ‘controls’.

Comparison study 2 was to assess the risk of readmission. The design of this study was similar to a retrospective cohort study. The study population was all patients who had at least one admission during the year surveyed. The entry point was defined as the index admission (the first admission during the year surveyed) and the end point was defined as the event of readmission or 30 June 2006 if there was no readmission following the index admission. The exposures were defined as the variables under study, and outcome was defined as the event of readmission. All patients were retrospectively followed up for at least 12 months. A total of 178 patients were included in this study.

### Statistical analysis

The distribution of the main outcomes (LOS, interval between each admission, time to readmission) was not in a normal distribution, but skewed. The data were analysed by using non-parametric statistics. The variables under investigation included both categorical and continuous variables. In choosing the statistical methods, these facts were taken into consideration. The data were analysed by using SPSS version 19.0 [22].

In the LOS study, the dependent variable (LOS) was transformed to a binary proportion, either case or control. The explanatory variables were predominantly categorical, but some were continuous variables. Four levels of statistical analyses were conducted. Descriptive statistics were used to assess the distribution of variables. Significance tests were used to identify the associations between each variable and the outcome. For categorical variables, the chi square test was used. For continuous variables, the Mann-Whitney U test was employed. Odds ratio calculation was used to estimate the magnitude of any association. Logistic regression test was used to estimate odds ratios whilst adjusting for confounders. Variables with a p value equal to or less than 0.1 were put through the regression test.

In the risk of readmission study, the data were censored data. The dependent variable was ‘life table survival’ (the event of readmission). The explanatory variables were both categorical and continuous variables. Four levels of statistical analyses were conducted. Descriptive statistics were used to assess the distribution of variables among the compared groups. The Kaplan-Meier life table method was used to estimate the risk of readmission. Log-rank test was used to compare the risk of readmission between different exposure statuses. Cox proportional hazard model regression test was used to estimate the relative risk whilst controlling potential confounders. Variables with a p value equal to or less than 0.1 were put through the Cox regression test.

## Results

### Socio-demographic characteristics of the sample

The mean age of our sample was 39 years old (SD = 11.15); 60% of patients were between 30 to 49 years old; 62% were men; 29% of patients were born overseas, but 43% patients identified as of non-Australian descent and 65% of them were Southern Europe and Middle East origin; 78% of patients were unemployed; 73% were either separated or never married; 18% of patients lived alone and 61% lived with family or relatives; 20% of patients lived in marginal accommodation, including 7% homeless; 69% of patients did not complete high school.

### Clinical characteristics of the sample

Forty-six per cent of patients had a psychiatric history of more than 10 years duration and 71% had previous psychiatric admissions; 22% of patients had a forensic history. Nearly 52% patients had previous history of self-harm or suicidal attempts and 44% had previous history of aggression towards others; 57% of patients were admitted as involuntary patients, but only 32% were discharged on a community treatment order (CTO); 48% of patients were case managed by NWAMHS prior to the admission, but 61% were discharged to case management; 13% of patients required a new accommodation upon their discharge.

The primary reason for admission was potential risk issues in 84% admissions, including 18% admissions that followed a suicidal or self-harm attempt and 11% admissions that followed incidents of physical aggression towards humans or properties. On admission, 61% of patients were agitated, 47% of patients were suicidal and 11% of patients were homicidal.

The most common diagnosis was psychosis (60%), including 36% schizophrenia and 16% schizoaffective disorder (Tables 1 and 2). Substance abuse overall was very significant in this sample population, 59% had at least one drug or alcohol-related diagnosis (Table 3) and 30% of admissions were directly related to either drug intoxication or withdrawal. Twenty-nine per cent of patients had a diagnosis of personality disorder, including 19% borderline personality disorder.

**Table 1:** Principal diagnosis on admission

Diagnosis	N (226)	Percent (%)
Psychosis	136	60.2
Depression: unipolar	52	23.0
Bipolar disorder	26	11.5
Anxiety disorder	7	3.1
Somatoform disorder	7	3.1
Situational crisis	26	11.5
Adjustment disorder	14	6.2
Pathological gambling	3	1.3

Patients may have more than one Axis-I disorder.

**Table 2:** Types of psychotic illness

Diagnosis	N (136)	Percent (60.2%)
Schizophrenia	82	36.3
Schizophreniform	2	0.9
Schizoaffective disorder	35	15.5
Delusional disorder	6	2.7
Shared psychotic disorder	1	0.4
Drug induced	7	3.1
Not otherwise specified (NOS)	3	1.3

**Table 3:** Drug/alcohol (D/A)-related diagnosis

Diagnosis	N (226)	Percent (%)
D/A-related disorder	133	58.8
Alcohol use disorder	92	40.7
Acute alcohol disorder	35	15.5
Poly-substance misuse	88	38.9
Amphetamine misuse	69	30.5
Amphetamine intoxication	13	5.8
Cannabis misuse	107	47.3
Cannabis intoxication/withdrawal	21	9.2

D/A related diagnoses are mutually inclusive, a patient can have multiple D/A related diagnoses.

### Length of stay (LOS)

The mean duration of hospitalization was 15 days (SD = 13.55), with a range of 1 to 85 days. The median number of days of hospitalization was 12. Thirty-eight per cent of patients were discharged within 7 days of admission and 85% within 28 days. There were two peaks of LOS, which accounted for 55% of all admissions. The first peak of LOS was between 2 to 6 days (N = 73, 32%) and the next peak was between 10 to 15 days (N = 50, 22%). We also looked at the primary reasons for further hospitalization at different times. Potential risks to self or others were the main reason for further hospitalization at day 3 and day 7. At day 10 and day 14, logistic reasons became the main reasons for further hospitalization. Logistic reasons included lack of social and family support, lack of accommodation, negotiation with community team, and other practical difficulties interfering with discharge. At day 28, compliance issues became the main reason for further hospitalization.

Sixteen variables had a p value equal to or less than 0.10. Logistic regression analyses showed that 10 variables were associated with LOS. Three variables predicted longer stay and 7 variables predicted shorter LOS (see Table 4). Seclusion, accommodation problems and living in the Broadmeadows catchment area predicted longer stay. Variables that predicted a shorter stay were being a migrant from non-western countries (but not English proficiency), having completed high school, having a drug or alcohol-related diagnosis, admission directly related to a crisis, adjustment disorder, cluster B personality disorder, and having recently transferred care from other mental health services. Several variables thought to be powerful predictors of LOS were found not to be so. They included living alone, living in marginal accommodation, LOS of previous admission, chronicity of illness, diagnosis of schizophrenia or other psychotic illness, severity of illness, risk on admission. Also, the quality of inpa-tient or community care did not influence LOS: for instance, pharmacological intervention (including rapid tranquillization, medication change and dosing methods, the use of benzodiazepine), frequency of review, carer involvement, having a current individual service plan (ISP), assertive treatment, case conference, crisis team involvement, case management.

**Table 4:** Results of logistic regression

Variables	P value	Exp (B)	95%CI
Non-western born	0.004	0.27	0.10-0.65
Completed high school	0.006	0.34	0.16-0.73
New accommodation	0.030	3.54	1.13-11.13
Seclusion	0.016	4.06	1.30-12.68
D/A related diagnosis	0.048	0.45	0.21-0.99
Situational crisis	0.027	0.19	0.04-0.83
Adjustment disorder	0.045	0.10	0.01-0.95
Cluster B personality disorder	0.043	0.38	0.15-0.97
Broadmeadows area	0.048	2.65	1.01-6.95
Transferred from out of area	0.044	0.20	0.04-0.96

### Risk of readmission

A total of 82 patients (46%) were readmitted during the follow-up period, including 78 patients (40%) who had a readmission within 12 months of the index discharge; 23 patients (13%) had at least two readmissions within 12 months; 14 patients (8%) had three or more readmissions within 12 months.

Thirteen variables had a p value equal to or less than 0.1. Cox regression analyses showed nine variables were related to the risk of readmission (see Table 5). Six of these variables increased the risk of readmission. They included: the number of previous admissions, recorded deterioration of mental state prior to the index admission, risk to others at the time of index admission, contact with emergency department post discharge, alcohol intoxication on index admission, and electro-convulsive therapy (ECT) during the index admission. More proactive and assertive treatment in the community post discharge decreased the risk of readmission: for instance, involuntary treatment in community, reviewing the individual service plan and transferring care to a new treating team. Patients' socio-demographic characteristics, a diagnosis of a major psychiatric illness, LOS, or the clinical practice and care provided at the inpatient unit during the index admission did not influence the risk of readmission.

**Table 5:** Results of Cox regression test

Variables	P value	Exp (B)	95%CI
Previous admission <2	0.022	0.21	0.05–0.80
MSE deterioration	0.023	3.08	1.17–8.10
Post discharge ED contact	0.000	14.05	3.26–60.68
Legal status change post discharge	0.011	0.06	0.01–0.53
Change in treating team	0.025	0.33	0.12–0.87
Risk to others on admission	0.008	5.75	1.59–20.82
Alcohol intoxication	0.041	3.29	1.05–10.32
ECT	0.001	12.85	2.99–55.19
Post discharge ISP	0.017	0.33	0.14–0.82

## Discussion

This study is a comprehensive descriptive study, including over 200 patient, clinical, and system/practice variables. Our sample population was very much a marginalized population. A large proportion of patients were single middle-aged men, either first or second generation migrants. They were often unemployed, poorly educated and living with relatives. A significant proportion of our patients were experiencing a psychosis with a chronic course, and had a comorbid drug and alcohol disorder. The majority of admissions were unavoidable as patients were in an actively psychotic or manic state with significant risk to self or others and requiring involuntary admission. This is the first study to directly audit the reasons for further hospitalization at different time frames, which cannot be captured in a study comparing mean or median LOS. Eighty-four percent of admissions were for risk containment, but after day 6 of the admission, the main reason for continued hospitalization was logistic or compliance-related. After day 10, 80% patients could have been managed at a facility other than acute inpatient services. Therefore, our study supports the recent development of policies and funding to provide increased service provision as an alternative to inpatient care, including respite or step-down beds, day programmes, outreach teams to supervise medication, and supervised accommodation [4,5,23,24].

Most LOS studies examined the difference in mean, rather than median. LOS in acute psychiatric facilities is skewed; therefore the mean is less informative [25]. That is one of the main reasons why outcomes of LOS studies are not consistent [26]. One of the strengths of this study was to look into the differences concerning variable distribution between two sub-populations divided by the median.

Patients from the Broadmeadows area had longer LOS. This is likely to be more related to the lack of services in the northern part of the catchment area rather than social disadvantage or inadequate case management, as these latter factors did not independently influence the LOS. Patients who required seclusion during hospitalization tended to have prolonged LOS. This might relate to behavioural manifestations of illness resulting from a combination of various factors, rather than psychosis or drug and alcohol-related behaviour. According to our data, lack of appropriate and affordable supported accommodation has been a significant issue in this catchment area and contributes to bed blocking. This finding is consistent with other published studies. In a UK study, it was suggested that lack of suitable supported accommodation was one of the major reasons preventing discharge [5]. Similarly, availability of secure housing was reported to be an important factor enabling discharge of patients who had spent extended periods in several Queensland hospitals [27].

Other results were consistent with previous published data, e.g. a diagnosis of drug or alcohol disorder, or cluster B personality disorder was associated with shortened LOS [22,28]. Our study also suggested that a diagnosis of adjustment disorder or situational crisis was associated with shortened LOS. In our sample population, migrant status and completing high school predicted a shortened LOS, which has not been previously reported, although Arab cultural background predicted shorter cumulative length of stay in an Israeli case register study [29]. The majority of migrants in our sample were from Southern Europe and the Middle East. Victorian mental health service data has shown that a disproportionately higher number of community clients from migrant backgrounds were admitted to acute inpatient units suggesting that they may present to community services at a later stage of their disorder [30]. A possible explanation for this and the present findings is that factors such as family-orientated cultural values might make the family more tolerant of the behavioural disturbance of their ill relatives. A similar interpretation was advanced in the afore-mentioned Israeli study [29]. We also hypothesize that an adequate education level might reflect good coping skills and good premorbid functional level, both contributing to shorter inpatient stay. Contrary to anecdotal evidence that patients transferred from other area mental health services tended to block our inpatient beds, they actually had shortened LOS.

Most patients had a diagnosis of psychosis (60%) and the most common reason for admission was for risk containment. This might reflect the fact that public mental health services mainly provide services to people with severe mental illness and a psychiatric admission is often preceded by behavioural disturbance that could not be managed in the community. Contrary to the common assumption, our study does not suggest that a diagnosis of severe psychiatric disorder, like psychosis, chronicity of illness and severity of illness influence the LOS. It may reflect the shifting of the care of this patient population to community-based psychiatric services following deinstitutional-ization. Community arms of psychiatric services are more resourced and skilled in providing services to these patients in the modern era of mental health service delivery in developed countries [24,31,32].

It is controversial whether a shorter LOS is associated with an increased risk of readmission [33-36]. In our sample, a significant proportion of patients had readmission within 12 months of discharge, but the risk of read-mission is not associated with LOS or clinical factors or quality of inpatient care during the index admission, apart from ECT treatment. Therefore the quality of inpatient care does not influence the rate of readmission. It raises a question about the validity of using the rate of readmission as an outcome measure for inpatient care [15,35,37,38].

As recently reported for patients with bipolar I disorder [39], the number of previous admissions within the past 12 months predicted the risk of readmission. Therefore it will be useful to identify the characteristics of ‘frequent users’ of acute psychiatric inpatient services. Good practice following index discharge likely reduces the risk of readmission including revising the individual service plan (ISP), more assertive follow-up by instigating a community treatment order (CTO), good assessment and intervention in the community.

We also identified a sub-population that was at risk of readmission. They were patients who had multiple previous admissions, misused alcohol, had ED contact, posed risk to others on index admission, or had ECT during the index admission. Our study suggests that readmission is predictable and the risk should be able to be minimized through identifying this at-risk population and adopting good community practice in their ongoing care.

One of the limitations of this study is that it is a retrospective file audit with 9% missing data. The data collection instrument included more than 200 variables that might in turn have diluted the significance of the findings. In Australia, consultant psychiatrists have a pivotal role in managing inpatients and operating acute inpatient services. Clinical anecdotal evidence suggests that a psychiatrist's orientation influences the LOS. We did not measure the influence of psychiatrists' variables on the LOS. This study looked into association between migrant status and LOS and readmission, but did not study the difference between culturally and linguistically diverse (CALD) communities. The quality of both inpatient and community care was very much defined by clinicians rather than consumers' and carers' perspectives. These areas might warrant further study as suggested by others [15].

In conclusion, LOS is multifactorially determined. Behavioural manifestations of illness resulting from a combination of factors and lack of social support structures predict prolonged LOS. Good clinical practice does not necessarily translate to a shorter LOS. Therefore LOS is predictable, but not readily modifiable within the clinical domain. A sub-population of patients who requires frequent psychiatric admission is identifiable. The quality of inpatient care does not influence the risk of readmission, which therefore raises a question about the validity of using the rate of readmission as an outcome measure of psychiatric inpatient care. Good psychiatric practice within the community following discharge likely reduces the risk of readmission.
